# Advances in stem cell-based therapeutics for acute high-altitude illness: research progress and prospects

**DOI:** 10.3389/fphys.2025.1614098

**Published:** 2025-08-01

**Authors:** Bai-Tao Dou, Meng-Jiao Li, Yan-Ling Li, Dan Chen, Cheng-Wei Yang, Fang-Yi Fan, Hao Yao

**Affiliations:** ^1^ Department of Hematology, Chinese People’s Liberation Army The General Hospital of Western Theater Command, Chengdu, Sichuan, China; ^2^ Branch of National Clinical Research Center for Hematological Disease, Chengdu, Sichuan, China; ^3^ Sichuan Clinical Research Center for Hematological Disease, Chengdu, China; ^4^ Department of Clinical Medicine, North Sichuan Medical College, Nanchong, Sichuan, China; ^5^ Department of Hematology, Chongqing University Fuling Hospital, Chongqing, China; ^6^ Department of Orthopaedics, The 940th Hospital of Joint Logistics Support Force of PLA, Lanzhou, Gansu, China; ^7^ Institute of Basic Medicine, North Sichuan Medical College, Nanchong, Sichuan, China; ^8^ Tissue Stress Injury and Functional Repair Key Laboratory of Sichuan Province, Chengdu, Sichuan, China

**Keywords:** acute high-altitude illness, mesenchymal stem cells, neural stem cells, induced pluripotent stem cells, hypoxia adaptation

## Abstract

Acute high-altitude illness (AHAI), triggered by hypobaric hypoxia following rapid ascent to high elevations, induces complex pathophysiological responses that may be life-threatening. Recent advances in regenerative medicine have highlighted the therapeutic potential of stem cells in mitigating hypoxia-induced damage. Among them, Mesenchymal stem cells (MSCs), the most extensively investigated, exert therapeutic efficacy through immunomodulation, attenuation of oxidative stress, and enhancement of tissue repair mechanisms. Their paracrine signaling profile facilitates angiogenesis and stabilization of the hypoxic microenvironment. Neural stem cells (NSCs) exhibit robust proliferation and differentiation under hypoxic conditions, offering a novel avenue for the treatment of high-altitude cerebral pathology. Additionally, induced pluripotent stem cells (iPSCs), with their pluripotency and patient-specific derivation, present significant promise for personalized, cell-based interventions. Experimental studies demonstrate that these stem cell types modulate the hypoxic milieu via secretion of cytokines, remodeling of the immune microenvironment, and promotion of neovascularization. Nonetheless, several translational challenges persist, including suboptimal homing efficiency, potential immunogenicity, and uncertain long-term safety profiles in high-altitude settings. Future research should prioritize elucidation of stem cell behavior in hypobaric hypoxia, optimization of delivery systems, and establishment of standardized therapeutic protocols. Rigorous clinical validation through evidence-based approaches will be essential to substantiate safety and efficacy. With continued advances in stem cell biology and translational techniques, stem cell-based therapy is poised to emerge as a viable strategy for the prevention and management of AHAI, provided that its clinical deployment is underpinned by robust scientific evidence.

## 1 Introduction

High-altitude environments are defined by hypobaric hypoxia, and rapid exposure to such conditions without adequate acclimatization can induce a cascade of physiological responses such as dyspnea and headache, which may progress into life-threatening acute high-altitude illness (AHAI). This condition encompasses acute mountain sickness (AMS), high-altitude cerebral edema (HACE), and high-altitude pulmonary edema (HAPE), and can also exacerbate pre-existing cardiopulmonary disorders (Derstine et al., 2024).

Epidemiological studies have shown that approximately 25%–40% of unacclimatized individuals ascending above 2,500 m develop AMS, with a subset progressing to severe forms such as HAPE or HACE (Derstine et al., 2024). Although pharmacologic treatments are available, they often have delayed onset and limited efficacy in advanced stages, particularly for neurovascular complications (Ercan, 2021). These limitations underscore the need for novel therapeutic approaches such as stem cell therapy, which may offer broader protective effects through immunomodulation, vascular repair, and tissue regeneration.

Current therapeutic strategies include descent to lower altitudes, oxygen supplementation, and administration of pharmacologic agents such as acetazolamide, ibuprofen, dexamethasone, phosphodiesterase type 5 (PDE5) inhibitors (e.g., tadalafil, sildenafil), and β-adrenergic receptor agonists (e.g., salmeterol) (Luks et al., 2024). However, the early manifestations of AHAI are often non-specific, and once the disease progresses, drug interventions may have limited efficacy and significant adverse effects. Thus, identifying more effective and targeted prevention and treatment strategies is of critical importance.

Stem cells, defined by their self-renewal and multilineage differentiation capacities, are generally classified into embryonic stem cells, adult stem cells (including mesenchymal stem cells (MSCs) and neural stem cells (NSCs)), and induced pluripotent stem cells (iPSCs) based on their origin and differentiation potential (National Research Council, 2002). These cells exhibit immunomodulatory, anti-inflammatory, paracrine, and homing properties, enabling them to regulate inflammation, promote tissue regeneration, modulate immune responses, and, in some contexts, replace damaged cells (Phinney and Pittenger, 2017). In the field of AHAI research, adult stem cells such as MSCs, NSCs, and iPSCs have shown promising therapeutic potential, with MSCs being the most extensively investigated (Table 1). This review summarizes the current understanding of the mechanisms by which these stem cell types exert protective effects in AHAI, aiming to provide new perspectives for future research and clinical translation.

**TABLE 1:** Comparison of different stem cell types in the context of AHAI treatment.

Cell type	Main sources	Advantages	Limitations	Route of administration	Clinical application status	Potential side effects
Mesenchymal Stem Cells (MSCs)	Bone marrow, umbilical cord, adipose tissue	Readily accessible; low immunogenicity; strong immunomodulatory and tissue-repair properties	Limited neuronal differentiation capacity; mechanisms not fully elucidated	Intravenous injection, local injection, intratracheal delivery	Widely used in clinical studies across various diseases	Immune dysregulation, thrombosis, ectopic differentiation
Neural Stem Cells (NSCs)	Embryonic brain tissue, iPSCs	Capable of differentiating into neurons and glial cells; neuroprotective	Limited availability; ethical issues; limited clinical experience	Intrathecal injection, local delivery	Applied in preclinical and early-stage clinical neurological trials	Tumorigenicity, low post-transplant survival
Induced Pluripotent Stem Cells (iPSCs)	Reprogrammed somatic cells	Unlimited proliferation; multipotent; high potential for personalized therapy	Technically complex; genetic/epigenetic instability	Currently limited to experimental research	Used in disease modeling and mechanistic studies	Tumorigenicity, immune rejection, uncertain long-term safety

## 2 Research progress of MSCs in AHAI

### 2.1 Biological characteristics of MSCs

MSCs are a type of adult stem cell characterized by self-renewal and multipotent differentiation capacity. Initially discovered in bone marrow in the mid-20th century, subsequent studies have demonstrated that MSCs are also widely distributed in other tissues, including adipose tissue, umbilical cord, and dental pulp (Wang et al., 2022). A hallmark of MSCs is their ability to differentiate into multiple mesodermal lineages—such as osteoblasts, adipocytes, and chondrocytes—under specific culture conditions, rendering them highly valuable in regenerative medicine.

To ensure consistency across studies, the International Society for Cellular Therapy (ISCT) established minimal criteria for MSC identification in 2006 (Dominici et al., 2006). According to ISCT guidelines, MSCs must meet three core criteria: (1) adherence to plastic under standard *in vitro* conditions; (2) expression of surface markers CD105, CD73, and CD90, with negligible expression of hematopoietic markers (e.g., CD45, CD34) and MHC class II molecules; and (3) the capacity for trilineage differentiation into osteogenic, adipogenic, and chondrogenic cell types under specific inductive conditions. These criteria have provided a standardized framework for MSC-based research and clinical applications, significantly facilitating the development and reproducibility of the field.

#### 2.1.1 Mechanisms of MSCs in the prevention and treatment of AHAI

##### 2.1.1.1 Immunomodulation and anti-inflammatory effects of MSCs

**FIGURE 1:**
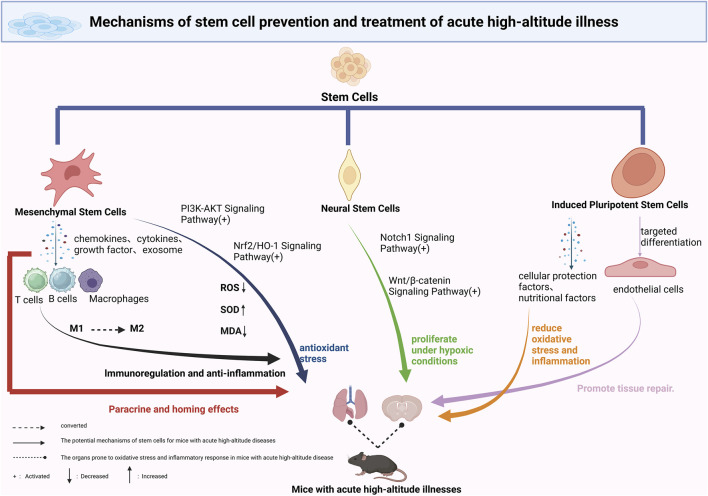
Mechanisms of Stem Cell-Based Therapies in Acute High-Altitude Illness. This schematic illustrates the proposed mechanisms through which various stem cell types contribute to the prevention and treatment of acute high-altitude illness. Mesenchymal stem cells (MSCs) exert anti-inflammatory and antioxidative effects via secretion of cytokines and exosomes. Neural stem cells (NSCs) may aid in the repair of hypoxia-induced neural damage through differentiation and modulation of the blood–brain barrier. Induced pluripotent stem cells (iPSCs) and their derivatives contribute through the release of protective paracrine factors, metabolic reprogramming, and delivery of exosomal miRNAs, enhancing tissue resilience to hypobaric hypoxia. Potential applications include reducing cerebral edema, improving pulmonary function, and restoring vascular integrity. Future strategies may involve combining stem cell therapy with conventional treatments to improve outcomes under high-altitude conditions.

MSCs exhibit a range of therapeutic mechanisms in the prevention and treatment of AHAI, with their immunomodulatory and anti-inflammatory effects being particularly prominent. MSCs release a variety of bioactive molecules, including cytokines, growth factors, and chemokines, through paracrine signaling. These molecules interact with immune cells, such as T lymphocytes, B lymphocytes, and macrophages, thereby playing an essential regulatory role in both innate and adaptive immune responses (Figure 1) (Gnecchi et al., 2016; Nauta and Fibbe, 2007). Hypoxic conditions at high altitudes induce significant inflammatory responses. Studies have shown that in environments above 3400 m, the levels of circulating inflammatory factors in healthy volunteers are markedly elevated, contributing to increased vascular permeability, fluid leakage, and the development of pulmonary or cerebral edema (Hartmann et al., 2000). MSCs can suppress inflammation by modulating macrophage polarization, promoting the conversion of pro-inflammatory M1 macrophages to anti-inflammatory M2 macrophages, thus maintaining tissue homeostasis (Aggarwal et al., 2014; Yin et al., 2018). Experimental research has confirmed that in low-pressure, hypoxic animal models, MSC-pretreated mice show a significant reduction in the proportion of M1 macrophages in alveolar lavage fluid, and a notable decrease in the mRNA expression levels of inflammatory factors such as IL-1β, IL-6, and TNF-α in lung tissues (Figure 1) (Yan et al., 2025).

Recent studies have further elucidated the specific molecular mechanisms underlying MSC-mediated immunoregulation, with MSC-derived extracellular vesicles (MSC-Exosomes) playing a pivotal role (Liao and Hsu, 2024). Exosomes are extracellular vesicles with a diameter of less than 150 nm, containing a variety of bioactive molecules, including proteins, nucleic acids, and lipids. They offer advantages such as low immunogenicity and high stability (Théry et al., 2018; Kim et al., 2025). MicroRNAs (e.g., miR-146a) enriched in MSC-Exosomes can regulate immune cell functions by modulating target gene expression. For example, human umbilical MSCs pretreated with IL-1β can transfer miR-146a via exosomes to macrophages, promoting their polarization to the M2 phenotype, thus enhancing anti-inflammatory effects (Song et al., 2017). Notably, hypoxic conditions significantly affect the biological properties of MSC-Exosomes. Research has shown that hypoxia not only increases the secretion of exosomes but also enhances their angiogenic potential through the activation of the HIF-1α signaling pathway (Li et al., 2022; Liu et al., 2020). In wound healing models, hypoxia-induced MSC-Exosomes upregulate miR-126 expression, promoting tissue repair (Sun et al., 2020). These findings provide new insights into the adaptive regulatory mechanisms of MSCs in high-altitude environments.

Recent progress in research has further expanded the potential applications of MSCs in AHAI prevention and treatment. A 2023 study demonstrated that stem cells derived from human exfoliated deciduous teeth (SHED) could effectively prevent HACE in a rat model by inhibiting the HIF-1α-mediated ERK signaling pathway, thereby regulating microglial polarization (Wang et al., 2023). Additionally, exosomes from miR-486-modified dental pulp stem cells exhibited better inflammatory regulation in high-altitude pulmonary edema animal models (Wang et al., 2025). These findings not only confirm the unique advantages of MSCs and their exosomes in hypoxic environments but also provide essential evidence for the development of novel therapeutic strategies. Compared to traditional drug treatments, MSC-based therapy offers multiple-target effects with fewer side effects, and its adaptive regulatory capacity in high-altitude environments may yield more favorable therapeutic outcomes. With continued research into MSC mechanisms and advancements in exosome technology, MSC-based interventions are poised to become an important therapeutic approach for the prevention and treatment of AHAI.

##### 2.1.1.2 Antioxidative stress mechanisms of MSCs in AHAI

MSCs exhibit significant therapeutic potential in combating oxidative stress, a property of considerable importance in the prevention and treatment of AHAI. Studies have demonstrated that bone marrow-derived MSCs can effectively treat various oxidative stress-related conditions, primarily by modulating multiple antioxidant enzyme systems. These include the expression of key antioxidant enzymes such as superoxide dismutases (SOD1, SOD2), catalase (CAT), and glutathione peroxidase (GPx) (Figure 1) (Valle-Prieto and Conget, 2010). When the human body is abruptly exposed to hypoxic conditions at high altitudes, it produces a large number of free radicals, leading to a sharp increase in oxidative stress. This state results in a cascade of pathological changes, including lipid peroxidation, protein denaturation, and DNA damage (Dosek et al., 2007). Experimental data have shown that within 1.5 h of acute hypoxic exposure, levels of reactive oxygen species (ROS) and the oxidative stress marker malondialdehyde (MDA) significantly increase, underscoring the rapid impact of the high-altitude environment on the body’s redox balance (Sarada et al., 2008).

In animal models of high-altitude pulmonary edema (HAPE), dental pulp-derived MSCs (DPSCs) have demonstrated notable antioxidative effects. Experimental results confirm that DPSCs can significantly enhance SOD activity and reduce MDA levels in lung tissue homogenates. This protective effect is likely mediated through activation of the nuclear factor erythroid 2–related factor 2/heme oxygenase-1 (Nrf2/HO-1) signaling pathway (Mao et al., 2024). As a key transcription factor in the oxidative stress response, Nrf2 plays a central role in regulating the expression of various antioxidant enzymes. Additionally, recent studies have found that extracellular vesicles derived from miRNA-486-modified DPSCs (DPSC-EVs) exert antioxidative effects via the PI3K-Akt signaling pathway, offering new perspectives on the antioxidative mechanisms of MSCs (Wang et al., 2025). These findings not only confirm the antioxidative protective effects of MSCs under hypoxic high-altitude conditions but also suggest that they may counteract oxidative damage through multiple signaling pathways.

Although research on the antioxidative stress effects of MSCs has made significant progress, the underlying mechanisms remain to be fully elucidated. The oxidative stress process involves complex enzyme systems and signaling networks, and MSCs may act through multiple coordinated pathways. Future studies should further investigate the antioxidative mechanisms of MSCs in different high-altitude illness models, including their effects on mitochondrial function, redox homeostasis, and apoptosis. Moreover, optimizing administration routes, dosage, and exploring combination therapies with MSCs could provide a solid foundation for developing more effective treatment strategies for high-altitude diseases. As our understanding of MSC-mediated antioxidative mechanisms deepens, their application prospects in high-altitude medicine will continue to expand.

##### 2.1.1.3 Key mechanisms of MSC paracrine function and homing in the prevention and treatment of AHAI

The therapeutic effects of MSCs in AHAI largely rely on their distinctive paracrine function and homing capability. MSC homing can be categorized into non-systemic and systemic modes. Non-systemic homing refers to the direct delivery of MSCs to the injury site through local transplantation, whereas systemic homing is more complex. It involves the directed migration of MSCs across the vascular endothelial barrier to target tissues under the guidance of chemokines released from injured areas (Nitzsche et al., 2017). Studies have shown that once MSCs enter the circulatory system, they preferentially accumulate in sites of inflammation and tissue damage, a property that provides a unique advantage in repairing injuries induced by high-altitude exposure (Sullivan et al., 2013).

This homing process is governed by intricate molecular mechanisms, among which the interaction between stromal cell-derived factor-1 (SDF-1) and its receptor C-X-C chemokine receptor type 4 (CXCR4) is particularly crucial. Upon tissue injury, locally released SDF-1 forms a chemotactic gradient that binds to CXCR4 expressed on the MSC surface, initiating intracellular signaling that guides MSC migration to the injury site (Xiao et al., 2016). In animal models simulating hypobaric hypoxia at high altitudes, this mechanism has been well validated: intravenously injected MSCs successfully homed to damaged lung tissue and significantly ameliorated pathological changes such as alveolar wall thickening, interstitial edema, and inflammatory cell infiltration (Yan et al., 2025; Wang et al., 2025; Mao et al., 2024). Notably, the efficiency of MSC homing is positively correlated with the severity of tissue injury—greater damage leads to stronger chemotactic signaling and more effective MSC recruitment (Gnecchi et al., 2016; Wang et al., 2023; Sullivan et al., 2013).

The paracrine effect of MSCs complements their homing capability, together forming a critical defense mechanism against high-altitude-induced tissue injury. Once MSCs reach the damaged site, they secrete a variety of bioactive molecules, including growth factors, anti-inflammatory mediators, and antioxidants, thereby creating a therapeutic microenvironment (Gnecchi et al., 2016). These paracrine factors not only directly mitigate inflammation and oxidative stress but also activate endogenous repair mechanisms, promoting the restoration of tissue structure and function. In models of high-altitude lung injury, MSCs effectively interrupt the vicious cycle of inflammation and oxidative damage through this combined “homing–paracrine” mode of action, providing a theoretical basis for the development of novel treatment strategies for high-altitude illnesses, including potential improvements in MSC homing and paracrine functions.

### 2.2 Advances in research on NSCs in AHAI

#### 2.2.1 Fundamental characteristics of NSCs

NSCs are a type of adult stem cell characterized by their capacity for self-renewal and multipotent differentiation. In the adult brain, NSCs are primarily located in two specific regions: the subventricular zone (SVZ) of the lateral ventricles and the subgranular zone (SGZ) of the dentate gyrus in the hippocampus (Obernier and Alvarez-Buylla, 2019). These stem cells can differentiate into the three major cell types of the nervous system: neurons, oligodendrocytes, and astrocytes. Common markers for NSCs include the transcription factor Sox2, the surface antigen CD133, and glial fibrillary acidic protein (GFAP).

NSCs have shown considerable therapeutic potential in the treatment of neurological disorders. For instance, in a mouse model of Parkinson’s disease, NSCs transplantation has been demonstrated to significantly alleviate vascular injury and promote the recovery of neural function (Li et al., 2025).

#### 2.2.2 Regulatory mechanisms of hypoxia on NSCs

In 1992, Reynolds and colleagues successfully isolated NSCs from the striatum of adult mice, marking a new era in NSC research (R et al., 1992). Under hypoxic conditions, the biological behavior of NSCs is tightly regulated by various families of transcription factors, with hypoxia-inducible factors (HIFs) and nuclear factor kappa B (NF-κB) playing central roles. Studies have shown that HIF-1α modulates downstream target genes such as erythropoietin (EPO), vascular endothelial growth factor (VEGF), and key molecules in the Notch and Wnt signaling pathways, collectively influencing NSC proliferation, differentiation, and apoptosis (Fan et al., 2023).

Notably, the Notch1 signaling pathway is significantly activated under hypoxia. Not only does it regulate the balance between NSC self-renewal and differentiation but it also directly affects the survival of newly generated neurons (Koch et al., 2013; Mutoh et al., 2012). The Wnt/β-catenin signaling pathway has also garnered attention. *In vitro* experiments have demonstrated that hypoxic conditions enhance the stability of β-catenin and the expression of its downstream target genes (Figure 1), thereby promoting NSC proliferation, increasing survival rates, and enhancing differentiation potential (Braunschweig et al., 2015).

However, the effects of hypoxia on NSCs are evidently concentration- and time-dependent. Santilli et al. reported that an oxygen concentration range of 2.5%–5% is optimal for maintaining NSC proliferation and differentiation (Santilli et al., 2010). Animal studies further confirmed that intermittent hypoxia simulating altitudes of 3,000 m and 5,000 m significantly increases NSC proliferation in the brain, with BrdU-positive cell counts in the subventricular zone (SVZ) and dentate gyrus (DG) increasing by 62% and 35%, respectively (Zhu et al., 2005).

#### 2.2.3 Potential applications of NSCs in the prevention and treatment of HACE

When individuals rapidly ascend to high-altitude environments characterized by low pressure and hypoxia, they may develop HACE, a condition that poses a serious threat to life. Although there are currently no reports on the direct application of NSCs in HACE prevention or treatment, their established roles in central nervous system (CNS) injury repair suggest that NSCs may exert therapeutic effects through multiple mechanisms. Pathologically, magnetic resonance imaging (MRI) of HACE patients often reveals reversible white matter edema in regions such as the splenium of the corpus callosum—findings similar to the vasogenic edema observed in ischemic stroke (IS) (Hackett and Roach, 2004). Notably, NSC transplantation has demonstrated clear therapeutic benefits in IS animal models, providing a theoretical foundation for investigating their application in HACE (Obernier and Alvarez-Buylla, 2019; Li et al., 2025; R et al., 1992; Fan et al., 2023).

NSCs may alleviate the pathological progression of HACE through several pathways: first, transplanted NSCs can differentiate into functional neurons and glial cells, thereby replacing damaged neural components; second, NSCs secrete neurotrophic and anti-inflammatory factors via paracrine mechanisms, improving the local microenvironment; third, NSCs may help regulate the permeability of the blood–brain barrier (BBB), thereby reducing vasogenic cerebral edema. Importantly, the hypoxic conditions associated with high-altitude exposure can themselves stimulate the proliferation and differentiation of endogenous NSCs, offering a promising direction for developing NSC-based endogenous repair strategies.

### 2.3 Advances in the application of iPSCs in the treatment of AHAI

#### 2.3.1 Fundamental characteristics and application prospects of iPSCs

iPSCs are a type of pluripotent stem cell generated by reprogramming terminally differentiated somatic cells through the introduction of specific transcription factors. These cells possess the ability to differentiate into all somatic cell types derived from the endoderm, mesoderm, and ectoderm, offering broad potential in the field of regenerative medicine.

One of the major advantages of iPSC technology is its capacity to generate patient-specific stem cells. Through directed differentiation, disease-relevant functional cells can be obtained, which are not only useful for drug screening and toxicity testing but also provide an ideal cell source for personalized therapy. This “patient-matched” therapeutic strategy brings new hope for the treatment of various refractory diseases.

#### 2.3.2 Protective mechanisms of iPSCs in hypoxia-related diseases

iPSCs exhibit unique cytoprotective properties in hypoxia-related diseases through multiple mechanisms. Firstly, iPSCs secrete a variety of bioactive paracrine factors, including vascular endothelial growth factor (VEGF), hepatocyte growth factor (HGF), and insulin-like growth factor-1 (IGF-1). These factors act synergistically within complex signaling networks to significantly enhance tissue tolerance to hypoxia. Under hypoxic conditions, iPSCs also upregulate the expression of antioxidant enzymes such as superoxide dismutase (SOD) and catalase (CAT), effectively scavenging excess reactive oxygen species (ROS) and mitigating oxidative stress-induced damage. Studies have shown that iPSC-conditioned medium (iPSC-CM) contains abundant protective exosomes—nanoscale vesicles capable of delivering protective microRNAs (miRNAs) and proteins to injured cells, thereby modulating their gene expression profiles and metabolic states (Aldali et al., 2025; Bobis-Wozowicz et al., 2015).

At the molecular level, the protective effects of iPSCs involve the fine regulation of several key signaling pathways. In hypoxic microenvironments, iPSCs activate pro-survival pathways such as PI3K/Akt and ERK1/2, thereby inhibiting apoptotic processes. Concurrently, iPSCs significantly downregulate the expression of pro-inflammatory cytokines such as TNF-α and IL-6, alleviating the inflammatory response. Recent studies have revealed that exosomes derived from iPSCs can deliver protective miRNAs, including miR-21 and miR-210, which suppress the expression of pro-apoptotic proteins such as PDCD4 and Caspase-3 (Figure 1) (Jin et al., 2025; Wang et al., 2015).

Importantly, iPSCs undergo metabolic reprogramming under hypoxic conditions, shifting from oxidative phosphorylation to glycolysis. This metabolic switch not only enhances their survival but also boosts their paracrine protective functions. Additionally, iPSCs help maintain cellular homeostasis during hypoxic injury by modulating autophagic activity, playing a critical role in tissue repair.

#### 2.3.3 Potential applications of iPSCs in AHAI

Although direct studies on the application of iPSCs in AHAI are currently limited, their established efficacy in other hypoxia-related diseases suggests multiple potential therapeutic mechanisms. First, iPSC-derived conditioned medium is rich in various nutritional and cytoprotective factors, which may help mitigate oxidative stress and inflammatory injury caused by high-altitude hypoxia. Second, specific functional cells derived from directed iPSC differentiation—such as alveolar epithelial cells and vascular endothelial cells—may replace damaged tissues and promote organ recovery. Notably, iPSC technology enables the development of patient-specific therapeutic approaches, which is of great significance for high-altitude illness given the considerable inter-individual variability in susceptibility and response.

## 3 Discussion

Current research demonstrates that stem cell therapy holds significant potential in the prevention and treatment of AHAI. Among various types, MSCs have been most extensively studied due to their wide availability and ease of isolation. MSCs exhibit multiple therapeutic effects including anti-inflammatory, antioxidant, and tissue repair capabilities. NSCs and iPSCs also display unique proliferative and differentiation characteristics as well as cytoprotective effects under hypoxic microenvironments, providing novel therapeutic avenues for specific pathologies such as high-altitude cerebral injury. These findings collectively lay a critical foundation for developing innovative treatment strategies for AHAI. Although embryonic stem cells (ESCs) are fully pluripotent and have played a foundational role in stem cell biology, there is currently no research directly supporting their application in acute high-altitude illness. Therefore, the current study and most related research have focused on adult stem cell types such as MSCs, NSCs, and iPSCs, which have demonstrated more relevance in hypoxia-related conditions.

In addition to high-altitude illness, other hypoxic conditions such as prolonged air travel or spaceflight may involve similar mechanisms, including oxidative stress and neurovascular impairment (Ercan, 2021). Although less severe, these environments can still cause physiological strain. Given their anti-inflammatory and tissue-repair properties, stem cells may also have therapeutic potential in such contexts. Further research could help extend the application of SCT to a broader range of hypoxia-related conditions.

Despite these advances, numerous scientific challenges remain unresolved. At the basic research level, our understanding of stem cell biology in high-altitude-specific microenvironments is still limited. Key questions such as how differences in oxygen partial pressure at varying altitudes affect stem cell survival, homing, and differentiation efficiency, and whether hypobaric hypoxia alters the paracrine profile of stem cells, require further investigation. Standardized animal models of high-altitude illness and *in vitro* simulation systems are essential for addressing these fundamental issues.

Mechanistically, most current studies focus on phenotypic observations, with insufficient exploration of the underlying molecular regulatory networks. For example, the specific signaling pathways through which MSC-derived exosomes exert therapeutic effects remain incompletely understood; the neuroprotective mechanisms of NSCs in high-altitude brain injury are still under debate; and the genomic stability of iPSC-derived cells under high-altitude conditions warrants long-term evaluation. Moreover, differences in therapeutic outcomes and mechanisms among stem cells derived from various sources—such as bone marrow, umbilical cord, or adipose tissue—need to be systematically compared.

Translational application also faces multiple challenges. First, standardized protocols for the preparation and quality control of stem cell products remain to be established, particularly concerning how to maintain cell viability and therapeutic efficacy in high-altitude settings. Second, optimal therapeutic strategies must be defined by considering multiple factors: Should intervention be preventive or administered post-onset? Should delivery be local or systemic? How should cell dosage be personalized according to altitude and disease severity? These questions must be validated through rigorous clinical trials.

Safety concerns must not be overlooked. In high-altitude environments, the body is under stress, raising questions about whether stem cell therapy may increase the risk of thrombosis or immune dysregulation. Although long-term follow-up studies indicate a favorable safety profile for stem cell therapy in conventional diseases, there remains a lack of safety data specific to high-altitude conditions. Ethical issues, particularly those related to gene modification technologies such as iPSCs, also require cautious consideration.

Future research should explore several key directions regarding iPSCs: (1) Establishing iPSC-based disease models specific to high-altitude illness to simulate cellular responses under varying altitudinal conditions; (2) Optimizing iPSC differentiation protocols to obtain functional cells with enhanced high-altitude adaptability; (3) Developing iPSC-derived therapeutic delivery strategies to improve treatment efficacy.

In addition, future research should prioritize: (1) development of novel stem cell-based therapeutics, such as genetically modified stem cells and stem cell-derived exosomes, to enhance targeting and safety; (2) establishment of high-altitude disease-specific evaluation systems, including identification of new biomarkers and standardized outcome assessment criteria; (3) promotion of multicenter clinical trials to build an evidence base through evidence-based medicine; and (4) exploration of combined treatment strategies involving stem cells and other therapies, such as hyperbaric oxygen or pharmacological agents. With progressive resolution of these critical issues, stem cell therapy is expected to become an integral component of comprehensive strategies for managing AHAI.
